# *N*-Salicyloyltryptamine, an *N*-Benzoyltryptamine Analogue, Induces Vasorelaxation through Activation of the NO/sGC Pathway and Reduction of Calcium Influx

**DOI:** 10.3390/molecules23020253

**Published:** 2018-01-28

**Authors:** Robson Cavalcante Veras, Darizy Flávia Silva, Lorena Soares Bezerra, Valéria Lopes de Assis, Walma Pereira de Vasconcelos, Maria do Carmo Alustau, José George Ferreira de Albuquerque, Fabíola Fialho Furtado, Islania Giselia de Albuquerque Araújo, Fátima de Lourdes Assunção Araújo de Azevedo, Thais Porto Ribeiro, José Maria Barbosa-Filho, Stanley Juan Chavez Gutierrez, Isac Almeida Medeiros

**Affiliations:** 1 Department of Pharmaceutical Sciences, Federal University of Paraíba (UFPB), João Pessoa 58059-900, Brazil; islania.ltf@gmail.com (I.G.d.A.A.); jbarbosa@ltf.ufpb.br (J.M.B.-F); isac@ltf.ufpb.br (I.A.M.); 2 Postgraduate Program of Nutrition Science/CCS/Federal University of Paraíba (UFPB); lorena.sbezerra@gmail.com; 3 Department of Biorregulation, Federal University of Bahia (UFBA), Av. Reitor Miguel Calmon, S/N, Vale do Canela, Salvador 40110-902, Brazil; darizy.silva@ufba.br; 4 Postgraduate Program of Natural Products and Bioactive Synthetics/CCS/Universidade Federal da Paraíba (UFPB), João Pessoa 58059-900, Brazil; val_farm@ltf.ufpb.com (V.L.d.A.); walmasjp@hotmail.com (W.P.d.V.); maria.alustau@ufcg.edu.br (M.d.C.A.); jgeorge_farm@msn.com (J.G.F.d.A.); fabiola.fialho@gmail.com (F.F.F.); fatima@ltf.ufpb.br (F.d.L.A.A.d.A.); thaispribeiro@hotmail.com (T.P.R.); stanleychavez@ufpi.edu.br (S.J.C.G.)

**Keywords:** vascular, calcium, nitric oxide, endothelium, rats, mesenteric

## Abstract

Benzoyltryptamine analogues act as neuroprotective and spasmolytic agents on smooth muscles. In this study, we investigated the ability of *N*-salicyloyltryptamine (STP) to produce vasorelaxation and determined its underlying mechanisms of action. Isolated rat mesenteric arteries with and without functional endothelium were studied in an isometric contraction system in the presence or absence of pharmacological inhibitors. Amperometric experiments were used to measure the nitric oxide (NO) levels in CD31+ cells using flow cytometry. GH3 cells were used to measure Ca^2+^ currents using the whole cell patch clamp technique. STP caused endothelium-dependent and -independent relaxation in mesenteric rings. The endothelial-dependent relaxations in response to STP were markedly reduced by L-NAME (endothelial NO synthase—eNOS—inhibitor), jHydroxocobalamin (NO scavenger, 30 µM) and ODQ (soluble Guanylyl Cyclase—sGC—inhibitor, 10 µM), but were not affected by the inhibition of the formation of vasoactive prostanoids. These results were reinforced by the increased NO levels observed in the amperometric experiments with freshly dispersed CD31+ cells. The endothelium-independent effect appeared to involve the inhibition of voltage-gated Ca^2+^ channels, due to the inhibition of the concentration-response Ca^2+^ curves in depolarizing solution, the increased relaxation in rings that were pre-incubated with high extracellular KCl (80 mM), and the inhibition of macroscopic Ca^2+^ currents. The present findings show that the activation of the NO/sGC/cGMP pathway and the inhibition of gated-voltage Ca^2+^ channels are the mechanisms underlying the effect of STP on mesenteric arteries.

## 1. Introduction

A healthy lifestyle is an important factor in prevention of diseases such as hypertension, diabetes and obesity [1]. However, in the cardiovascular area, a lifestyle modification may not always be expedient; therefore, medication is highly necessary, and the decision regarding therapeutic treatment should be based on the presence of risk factors, target-organ lesions and the presence of cardiovascular disease [1], most notably hypertension, coronary artery disease and heart failure [2]. 

Despite the considerable advances in pharmacotherapy for the management of hypertension over the past few decades, hypertension remains one of the major treatable epidemics in the world [3,4]. These limitations of the current therapies have stimulated the research and development of new classes of antihypertensive agents, mainly drugs that act on vascular endothelium and smooth muscle cells.

In particular, vasodilators are drugs that act by relaxing the smooth muscle walls of blood vessels and can reduce arterial pressure. Nitric oxide (NO) production can be stimulated by endogenous or exogenous agonists [5], in sequence the elevation of the cyclic GMP concentrations, reducing vascular resistance [6,7,8]. Other alternative to decrease vascular tonus is calcium-channel blockers (CCBs). They share the common property of blocking the transmembrane flow of calcium ions through voltage-gated L-type (slowly inactivating) channels [9]. These drugs display varying degrees of selectivity for different vascular beds and have distinct clinical profiles due to their particular mechanisms of action. 

CCBs have long been one of many possible antihypertensive therapeutics. One of the most puzzling characteristics of the CCBs is their chemical heterogeneity [10]. All calcium antagonists bind to the α-1c subunit of the L-type calcium channel, which is the main pore-forming unit of the channel [11], and relax vascular smooth muscle. As an in vivo correlate of these findings, CCBs block the responses of vascular smooth muscle to phenylephrine and High-K [9,12]. 

Pharmacological properties of tryptamine and its derivates are molecules with indolic ring involved in a large spectrum of biologic actions. In general, benzyl or phenethyl substituted tryptamines have significant effects on neuronal and gastrointestinal system [13,14]. A benzyltryptamine analogue, *N*-salicyloyltryptamine (STP), reduced the generation of action potentials in neuronal cells and strongly influencing neuronal excitability through its action on ion channels [15,16,17]. In guinea pig ileum, STP is a smooth muscle relaxant [18]. There are no studies of STP effects on cardiovascular system and few studies have explored this drug class in the literature. Therefore, the aim of this study was to evaluate the potential vasorelaxant activity of STP on mesenteric arteries to establish and elucidate its mechanism of action in this tissue.

## 2. Results

### 2.1. The Endothelium Participated in STP-Induced Relaxation 

**Figure 1:**
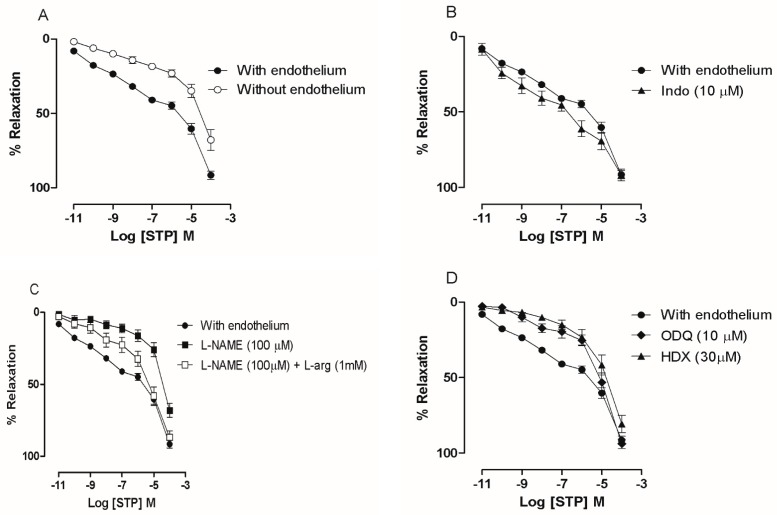
Vasorelaxant response of STP (0.01 nM–100 µM) in pre-contracted (Phe; 10 µM) superior mesenteric rings with (●, *n* = 11) or without endothelium (ο, *n* = 9) (**A**); In the presence of Indo (10 µM; ▲, *n* = 6) (**B**); In the presence of L-NAME (100 µM; ■, *n* = 9) (**C**); In presence of L-NAME (100 µM) plus L-arg (1 mM) (□, *n* = 6) (**D**); In the presence of HDX (30 µM; ▲, *n* = 8) or ODQ (10 µM; ♦, *n* = 8). The data are presented as the means ± SEM.

As shown in Figure 1, STP (0.01 nM–100 μM) induced concentration-dependent relaxation in pre-incubated (Phe, 10 μM) mesenteric artery rings with endothelium (pD_2_ = 6.30 ± 0.11, E_max_ = 91.6 ± 2.5%). The removal of the endothelium produced a rightward shift of the curve (pD_2_ = 5.58 ± 0.16, *p* < 0.05), with a significant reduction of the maximal effect (E_max_ = 68.0 ± 7.1%, *p* < 0.05) compared to the rings with intact endothelium (Figure 1A). 

Vasorelaxation was evaluated in the presence of several inhibitors to further investigate the endothelium-dependent mechanisms underlying the effects of STP. After treatment with L-NAME (pD_2_ = 4.98 ± 0.11, *p* < 0.05 and E_max_ = 68 ± 4.9%, *n* = 9, *p* < 0.05), the vasorelaxant effect of STP (0.01 nM–100 μM) was attenuated compared to the data obtained in the absence of inhibitors (Figure 1B). This suppression of the effects of STP by L-NAME was partially reversed by the addition of L-Arg (1 mM, pD_2_ = 5.78 ± 0.15), which partially restored the normal values of potency and the maximal total effect (87.0 ± 5.7%) compared to the rings with intact endothelium (Figure 1B). As shown in Figure 1C, HDX (30 µM) and ODQ (10 µM) caused a rightward shift of the STP concentration-response curve, thus reducing the pD_2_ to 5.37 ± 0.10 and 5.25 ± 0.11 (*p* < 0.05), respectively.

However, there was no significant modification of the maximal effect (94.0 ± 1.6% and 81.0 ± 5.7%, respectively). Together, the results suggest that the endothelium participates in the NO/sGC pathway.

### 2.2. NO Production 

**Figure 2:**
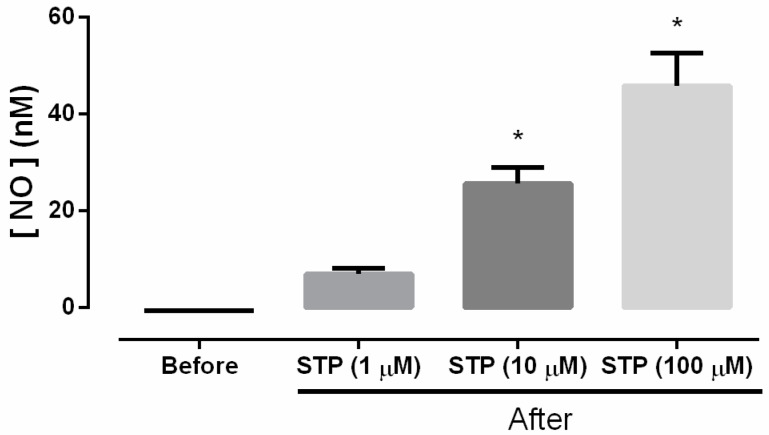
Representative bar graph of [NO] nM before and after the addition of STP (1, 10, and 100 µM) to selected samples of ECs; Data were normalized to the SNAP standard curve. The data are presented as the means ± SEM. Differences were analyzed by ANOVA One Way followed Bonferroni post-test. * *P* < 0.05 vs. Before

As shown in Figure 2, the results obtained with the amperometry technique and NO-selective microsensors showed that STP (10 and 100 µM) was capable of significantly increasing the NO concentrations in viable CD31+ cell suspensions (*p* < 0.05 vs. before).

### 2.3. Ca^2+^ Influx Attenuation Mediated Endothelium-Independent, STP-Induced Relaxation

**Figure 3:**
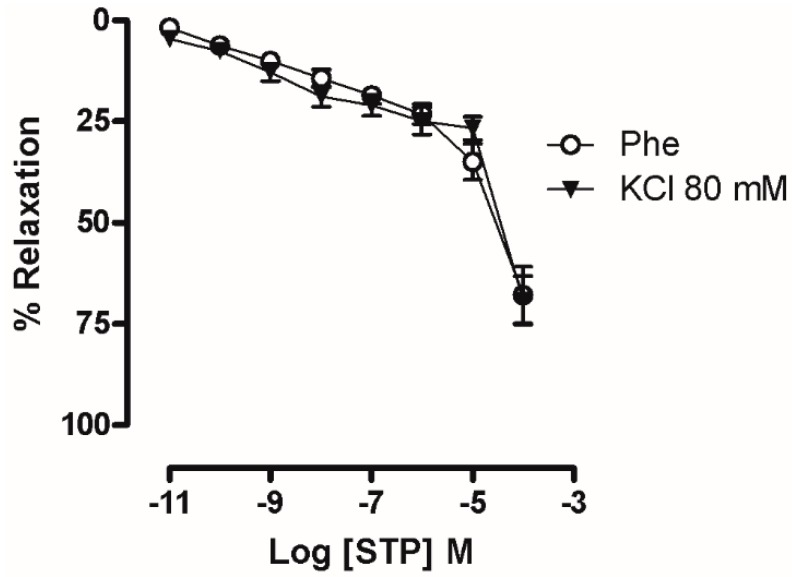
Vasorelaxant response of STP (STP; 0.01 nM–100 µM) in rings without endothelium pre-incubated with 80 mM KCl (▼, *n* = 9).

**Figure 4:**
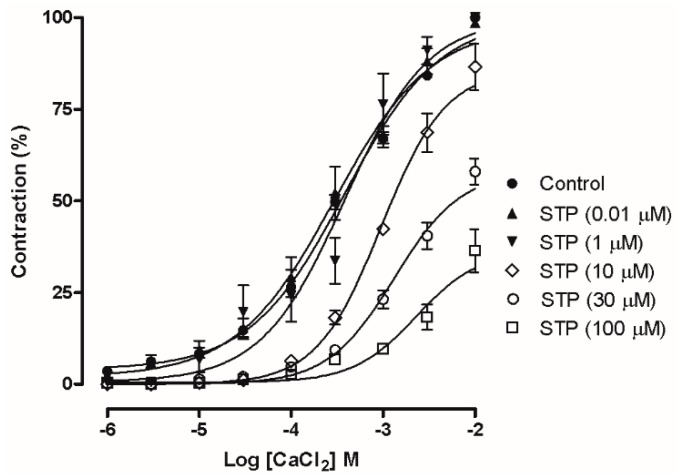
CaCl_2_ concentration-response curves of endothelium-denuded mesenteric artery rings in the absence (Control) or presence of STP (0.01 µM–100 µM). The data are presented as the means ± SEM.

In the denuded rings that had been incubated with KCl 80 mM (Figure 3A), the cumulative addition of STP induced a relaxation response (pD_2_ = 5.35 ± 0.18, E_max_ = 69.0 ± 6.0%) that was similar to the denuded rings that were pre-incubated with Phe (10 μM), with no significant differences. In addition, as shown in Figure 4, pretreatment with STP (0.01, 1, 10, 30 or 100 µM) attenuated CaCl_2_-dependent contraction in depolarizing medium. CaCl_2_ induced a concentration-dependent contraction, and pre-incubation with 10, 30 and 100 µM STP significantly reduced the E_max_ values (86.0 ± 6.2%, 58.0 ± 3.6%, and 36.5 ± 6.0%, respectively; *n* = 6 for each group), suggesting that the mechanism of action of STP involves the attenuation of Ca^2+^ influx.

### 2.4. Effect of STP on Ca^2+^ Currents

**Figure 5:**
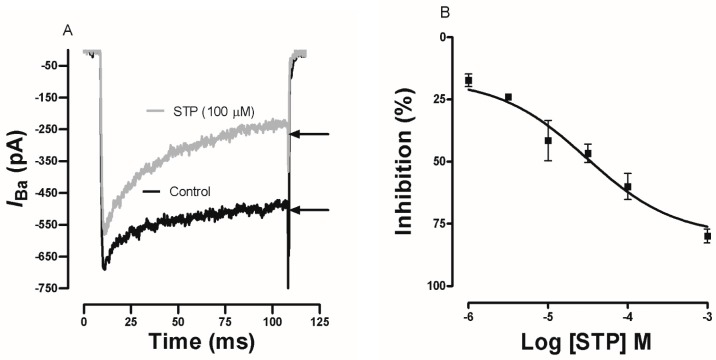
Effects of STP on the Ba^2+^ current in GH3 cells. (**A**) Typical recording of the Ba^2+^ current evoked by test pulses from −80 mV (holding potential) to 0 mV for 100 ms before perfusion with STP (control) and after perfusion with 100 µM STP. (**B**) Relationships between the Ba^2+^ current and STP concentrations. The data are presented as the mean values ± SEM.

Ca^2+^ currents through voltage-gated Ca^2+^ channels were evoked in GH3 cells by a depolarizing pulse to 0 mV (100 ms of duration) from a holding potential of −80 mV. Figure 5A shows the representative current traces obtained in the absence (Control) and in the presence of STP (100 µM). The STP (100 µM) perfusion reduced the inward Ca^2+^ current measured at the end of the pulse more than the current measured at the peak. Figure 5B shows the concentration-dependent relationship between the Ca^2+^ current at the end of the pulse and the drug concentration (1 µM–1 mM). The estimated pD_2_ was 4.53 ± 0.15. At the higher tested concentrations, STP inhibited approximately 80% of the Ca^2+^ currents, suggesting a possible effect on the voltage-gated Ca^2+^ channels.

## 3. Discussion

In this report, we investigated the vascular effects induced by STP in isolated mesenteric arteries. The major finding of this study was that this tryptamine analogue induced marked vasorelaxation by activating the NO/sGC pathway and reducing Ca^2+^ influx. The actions of STP have been investigated in some biological systems and have revealed the involvement of ionic channels [16,17,19]. However, there have been no studies reporting the effect of STP on the cardiovascular system. This report is the first to evaluate the vascular mechanisms of action of STP. In mesenteric arteries, STP induced marked, concentration-dependent relaxation, which was significantly reduced but not abolished after endothelium denudation, indicating that STP had endothelium-dependent and endothelium-independent effects (Figure 1A).

The endothelium plays an essential role in the control of vascular tone and is an important source of vasorelaxing factors, including prostacyclin (PGI_2_), nitric oxide (NO), and endothelium-derived hyperpolarization (EDH), which varies among different types of blood vessels [20,21,22,23,24]. The vasorelaxant response to STP was not mediated by cyclooxygenase metabolites of arachidonic acid (Figure 1B). Therefore, the endothelium-dependent effect seems to use another mechanism. 

In endothelium, NO is formed through constitutive NOS activity [25,26]. Pre-incubation with a competitive NOS inhibitor [25] showed that the vasorelaxant response of STP was significantly reduced to values similar to those observed in the endothelium-denuded preparations. The inhibition was partially reversed when the substrate of NOS, L-Arg, was previously added (Figure 1C), suggesting that STP could act on the endothelium to stimulate endothelial NO synthesis. The involvement of NO was reinforced by experiments with NO-scavenger (HDX) in which the STP-induced vasorelaxant response was significantly reduced (Figure 1D). Classically, HDX is described in the literature as an inhibitor of the biological effects of NO on vascular smooth muscle cells [27]. Under aerobic conditions and at physiologic pH, HDX is rapidly converted to HDX-O_2_^−^ (HDX-superoxide) and is spontaneously regenerated to HDX; this oxidative-reductive reaction leads to the rapid inactivation of NO [27,28]. Furthermore, regarding the vascular reactivity results, biochemical experiments using amperometric measurements showed that NO participated in vascular reactivity, where specific NO sensors detected a significant increase in NO concentrations after the cumulative addition of STP in freshly isolated EC suspensions (Figure 2).

NO acts on vascular smooth muscle through the pivotal activation of sGC, the primary mechanism by which NO induces relaxation, and has important pharmacological implications for the therapy of cardiovascular diseases in the cardiovascular system [29]. This action leads to an increase in guanosine 3,5-cyclic monophosphate (cGMP) concentrations and to the subsequent activation of cGMP-dependent protein kinase (PKG) and other effector molecules that can mediate cGMP-dependent relaxing effects in vascular smooth muscle cells (VSMCs) [30,31,32]. We observed that in presence of ODQ, an inhibitor of sCG, the concentration-response curve was shifted to the right (Figure 1D), helping to clarify the endothelium-dependent vasorelaxant mechanism of STP. These data from the global analysis showed that STP can increase NO production in endothelial cells and activate sGC to promote endothelium-dependent vasorelaxation.

In addition to its endothelium-dependent actions, STP had significant endothelium-independent activity. Several ion channels had fundamental importance in the vascular bed to maintain the tonus. The action of Ca^2+^ channels blockers is responsible for decreasing the peripheral resistance and have been used to reduce systemic arterial pressure. Furthermore, data from the literature indicate that STP is capable of blocking voltage-gated Ca^2+^ channels, inhibiting its currents in GH3 cells [16].

The participation of these channels in the STP endothelium-independent response was initially suggested by the results obtained in mesenteric rings that had been pre-incubated with a high K^+^ solution (Figure 3). Under this condition, STP induces vasorelaxation similar to the values obtained in rings contracted with Phe. That response can be obtained when Ca^2+^ influx is reduced [33,34]. In addition, STP was able to reduce the Ca^2+^ influx-dependent contractions in a concentration-dependent manner (Figure 4). These functional experiments showed that Ca^2+^ channel blockade participated in the STP response.

In corroboration, the electrophysiological experiments showed that STP inhibited the macroscopic Ca^2+^ currents in GH3 cells in a concentration-dependent manner. However, interestingly, our experiments have shown, for first time, that STP provoke a time-dependent inhibition of the Ca^2+^ current with an enhanced current decay during the voltage step (Figure 5a,b). All these findings fortify the hypothesis that STP acts on Ca^2+^ channels by diminishing the influx of this important ion in the contractile mechanism. The distrust in the use of GH3 cells as a model for the electrophysiology experiments was converted to confidence because cloning experiments [35] indicate that the pore of the L-type Ca^2+^ channel that is formed by the α1C subunit (or Cav1.2) in myocytes is also expressed in GH3 cells as a component of the high voltage-activated Ca^2+^ currents of the Cav1.2 channel [36,37]. This particular point is very important because the data had suggested the possible participation of the Ca^2+^ channels blockade in the vasorelaxant effects of STP, and these results support our main findings. 

## 4. Material and Methods

### 4.1. Animals

All animal care and experimental procedures were performed in accordance with Brazilian federal law No. 11794/08, which establishes the procedures for the scientific use of laboratory animals and institutional guidelines and Animal Care and Use Committee of the Federal University of Paraiba (CEPA#1003/07). Male Wistar rats (250–300 g) were used for all experiments. The animals were housed under a controlled temperature (21 ± 1 °C) and light cycle (lights on: 06:00–18:00 h). In addition, the animals had free access to water and food (Labina^®^, PURINA, São Paulo, Brazil). 

### 4.2. Drugs and Solutions

STP was kindly provided by Barbosa-Filho and colleagues (UFPB, João Pessoa, PB, Brazil) and was obtained using previously reported methods [18,38]. The drugs used were l-phenylephrine chloride (Phe), acetylcholine chloride (ACh), *N*^w^-nitro-l-arginine methyl ester (L-NAME), l-arginine, 1*H*-[1,2,4]oxadiazolo[4,3-*a*]quinoxalin-1-one (ODQ), CsCl, CsOH, ethylene glycol tetraacetic acid (EGTA), adenosine triphosphate-Mg (ATP-Mg), tetraethylammonium chloride (TEA), 4-(2-hydroxy-ethyl)-1-piperazineethanesulfonic acid (HEPES), tetrodotoxin (TTX) from Sigma-Aldrich^®^ (São Paulo, Brazil), and hydroxocobalamin from Cristalia (São Paulo, SP, Brazil). All drugs were dissolved in distilled water, except for ODQ, which was dissolved in DMSO.

### 4.3. Vascular Reactivity Studies 

The superior mesenteric artery (first branch) was removed from male Wistar rats (250–300 g), cleaned of connective and fat tissues in Tyrode’s solution (composition in mM: NaCl, 158.3; KCl, 4.0; CaCl_2_, 2.0; MgCl_2_, 1.05; NaH_2_PO_4_, 0.42; NaHCO_3_, 10.0 and glucose, 5.6) and segmentally cut into rings (1–2 mm). In some experiments, the endothelium was removed by gently rubbing the intimal surface of the rings with a thin wire.

The isometric tension was recorded using the method described by Veras et al. (2013). Briefly, the rings were suspended in an organ bath with 10 mL of Tyrode’s solution (pH 7.4) maintained at 37 °C and gassed with a 95% O_2_ + 5% CO_2_ mixture (pH 7.4). The rings were stabilized with an optimal resting tension and allowed to equilibrate for 60 min, with changes of Tyrode’s solution every 15 min.

The presence of functional endothelium was assessed by the ability of acetylcholine (ACh; 10 µM) to induce a >85% relaxation of vessels that had been pre-incubated with phenylephrine (Phe, 10 µM). The absence of a relaxation to ACh (<10%) was taken as evidence that the vessel segments were functionally denuded of endothelium [39]

The preparations were exposed to indomethacin (10 μM), a COX inhibitor; to L-NAME (100 μM), a nitric oxide synthase (NOS) inhibitor [40]; l-arginine (100 μM), an endogenous substrate of NOS [5]; hydroxocobalamin (HDX, 30 µM), an NO scavenger [28]; or ODQ (10 μM), a soluble guanylate cyclase (sCG) inhibitor [41,42]. All inhibitors were added 30 min before the application of Phe (10 µM) until the end of experiment. In the tonic phase of the second contraction, STP was cumulatively added (0.01 nM–100 µM) to the preparations until a maximum plateau response was observed.

The relaxation mechanism of STP was also evaluated using 80 mM KCl as contractile agent; in this case, the modified Tyrode’s solution (from 4 to 80 mM of KCl) was prepared by replacing Na^+^ with an equimolar concentration of K^+^ to maintain a constant ion strength in the bath solution [43].

Furthermore, CaCl_2_ concentration-response curves were generated in rings without endothelium to investigate the participation of calcium influx in STP-induced relaxation. Briefly, the CaCl_2_ concentration-response curves were constructed according to the method reported by Lagaud et al. [44], in which the tissues were initially pre-incubated with 60 mM KCl. After washing, Tyrode’s solution was replaced with Ca^2+^-free Tyrode’s solution. In addition, this solution was replaced with a Ca^2+^-free depolarizing solution (60 mM Ca^2+^-free KCl). CaCl_2_ concentration-response curves (1 µM–10 mM) were constructed in the absence or presence of STP (0.01, 1, 10, 30, and 100 µM), and the contractile responses were calculated as a percentage of the KCl contraction.

### 4.4. NO Measurement by Amperometry in Endothelial Cells (ECs)

#### 4.4.1. EC Isolation

Aortas were isolated, longitudinally opened, and cleaned. Endothelial cells (ECs) were mechanically isolated from the vessels by gentle friction with a plastic cell scraper in plates containing Tyrode’s solution. The cell pellet was washed twice, suspended (~10^6^ cells mL^−1^) in HEPES medium with 0.5% FCS (Invitrogen), and maintained in a humidified incubator (37 °C) until use [45,46,47]. Part of this cell suspension was used to detect and quantify the viable ECs using flow cytometry. 

#### 4.4.2. EC Detection and Viability

A FACSCanto II flow cytometer (Becton Dickson, San Jose, CA, USA) equipped with an argon laser (λex: 488 nm) was used to quantify cellular fluorescence. In each tube, 10,000 events were collected. Potential non-cellular particles were removed from the data with gates selected in the dot-plot graph of forward scatter (FSC) versus side-scatter (SSC). 

A non-stained aliquot was used as a negative control. In sequence, tubes with 10^6^ cells mL^−1^ were incubated with 1 µg mL^−1^ anti-CD 31-rat endothelium-PE antibody (OX43, sc53109, Santa Cruz Biotechnology, San Jose, CA, USA) for 45 min at 4 °C in the dark [48]. After washing, centrifugation (400× *g* for 5 min) and fixation, the cells were resuspended in HEPES medium and collected on a flow cytometer. The positively stained population in PE-channel (Low pass: 556 nm, λem: 485–42 nm) was considered CD31-positive cells [45]. 

Approximately 10^6^ cells mL^−1^ were incubated with 7-amino-actinomycin D (7-AAD, 5 µL) for 30 min at 4 °C in the dark to assess viability. The viable cells were observed in a dot-plot graph of PerCP (Low pass: 655 nm and Bandpass Filter: 670 nm) versus SSC (See Figure S1) [49]. 

#### 4.4.3. NO Determination by Amperometry

Cells suspensions with more than 75% viable, CD31-positive cells (See Figure S1) were used for the amperometric measurements of NO with ISONO microsensors (ISSO-NOP3005, World Precision Instruments, Inc., Sarasota, FL, USA). 

The NO meter was connected to a data acquisition system (TBR 4100—Free Radical Analyzer, World Precision Instruments, Inc., Sarasota, FL, USA) and to a PC with DataTrax-2 software (World Precision Instruments, Inc., Sarasota, FL, USA), through LABTRAX Bridge (World Precision Instruments, Inc., Sarasota, FL, USA). As previously reported by Zhang and colleagues [31], the sensors were calibrated by measuring *S*-nitroso-*N*-acetyl-d,l-penicillamine (SNAP, from World Precision Instruments, Inc., Sarasota, FL, USA) decomposition with CuCl_2_ as the catalyst [50,51]. SNAP is a nitrosothiol with the generic structure of RSNO. Under these conditions, the compounds decompose to liberate NO [52,53]. Initially, the NO sensor was immersed in 10.0 mL of the saturated CuCl_2_ solution, and the background current was allowed to decay to a stable value (150–3500 pA). The standard curve was obtained by the addition of SNAP into the CuCl_2_ solution (0.1 M) and constructed by plotting the amperage versus the concentration of NO liberated from SNAP [51]. In sequence, the cell suspension was transferred to the NO chamber (NOCHM-4, World Precision Instruments Inc., Sarasota, FL, USA) coupled to a previously calibrated NO sensor, and the NO concentration was measured before and after the addition of increasing concentrations of STP (1, 10 and 100 µM).

### 4.5. Electrophysiology

#### 4.5.1. GH3 Cell Culture

GH3 cells were obtained from the American Type Culture Collection and grown in HEPES-modified DMEM (Sigma, São Paulo, SP, Brazil) supplemented with 10% fetal bovine serum (Cultilab, São Paulo, SP, Brazil) and antibiotics (penicillin and streptomycin from Sigma-Aldrich, São Paulo, SP, Brazil). The cells were routinely grown as stocks in 75 cm^2^ flasks, as previously described [16]. The medium was changed twice a week. For the electrophysiological recordings, the cells were subcultured on glass coverslips and plated in 47 mm dishes.

#### 4.5.2. Electrophysiological Studies

Cultured GH3 cells were used in all experiments. The internal pipette solution contained: 130 mM CsCl, 20 mM TEA-Cl, 10 mM EGTA, 2 mM MgCl_2_, 4 mM ATPMg, and 10 mM HEPES. The pH was adjusted to 7.2 with CsOH. The external solution contained: 130 mM CsCl, 20 mM BaCl_2_, 0.5 mM MgCl_2_, 10 mM HEPES and 5 mM glucose. The pH was adjusted to 7.4 with CsOH. The external solution contained 100 nM tetrodotoxin to block the TTX-sensitive voltage-dependent Na^+^ channels. The pipettes were pulled on a PP-83 two-stage puller (Amityville, NY, USA) using glass capillaries (Perfecta, São Paulo, SP, Brazil) and had resistances of 2–4 MΩ when they were filled with the pipette solution. Only isolated cells were selected for recording to minimize space-clamp problems. The cells were not used for the recordings if the initial seal resistance was <2 GΩ.

An HEKA-EPC 9 amplifier with pulse, pulse-fit acquisition and analysis software (Instrutech, Longmont, CO, USA) was used to measure the whole-cell Ca^2+^ channel currents, which were recorded with Ba^2+^ as the charge carrier. Cancellation of the capacitance transients and leak subtraction was electronically performed using a P/4 protocol. Ca^2+^ currents were low pass filtered at 3 kHz and sampled at 10 kHz. The holding potential used to measure I_Ca_ was −80 mV. The test pulses of 0 mV were applied for 100 ms every 10 s. 

Series resistance was compensated by 50 to 60%. Patch-clamp experiments were performed in 47 mm Petri dishes using an inverted microscope (Olympus, New Hyde Park, NY, USA) with a 40× phase contrast objective. The bath was continuously perfused at 1–2 mL min^−1^ throughout the experiment. The solutions were gravity fed into the input ports of a solenoid valve mounted close to the bath, which was used to choose between one of two solutions. L-type Ca^2+^ currents were measured at the end of depolarization test pulse and plotted as a function of time (s).

### 4.6. Data Analysis

The values were expressed as the means ± SEM Contractile responses were expressed as a percentage of the maximal contractile response to 10 μM Phe or 80 mM K^+^ observed prior to the administration of any drug. Statistical significance was determined by *t*-test. ANOVA with repeated measures, followed by Bonferroni post-test, was used to analyze the Ca^2+^ influx blockade experiments. The pD_2_ was calculated using non-linear regression. All calculations were performed using GraphPad Prism© software, version 5.0 (GraphPad Software Inc., La Jolla, CA, USA). *p*-values < 0.05 were considered statistically significant. 

## 5. Conclusions

The present study showed that STP exerts an endothelium-dependent effect via NO release and an activation of the NO-sGC pathway using a combination of functional, amperometric and electrophysiological approaches. In addition, the mechanism of action of STP is also mediated by endothelium-independent activity that involves a reduction of Ca^2+^ influx and a blockade of voltage-gated Ca^2+^ channels. This report contributes to increase our knowledge about the actions of benzyltryptamine analogues, particularly in vascular tissue. In this regard, STP can be a valuable drug that acts on both the endothelium and smooth muscle.

## Supplementary Materials

The following are available online. Figure S1: Selection of Cells for NO measurement.
