# Supplementary figures and images for: N-Salicyloyltryptamine, an N-Benzoyltryptamine Analogue, Induces Vasorelaxation through Activation of the NO/sGC Pathway and Reduction of Calcium Influx
Source: Molecules. 2018 Jan 28;23(2):253. doi: 10.3390/molecules23020253 (PMC6017111; doi:10.3390/molecules23020253)

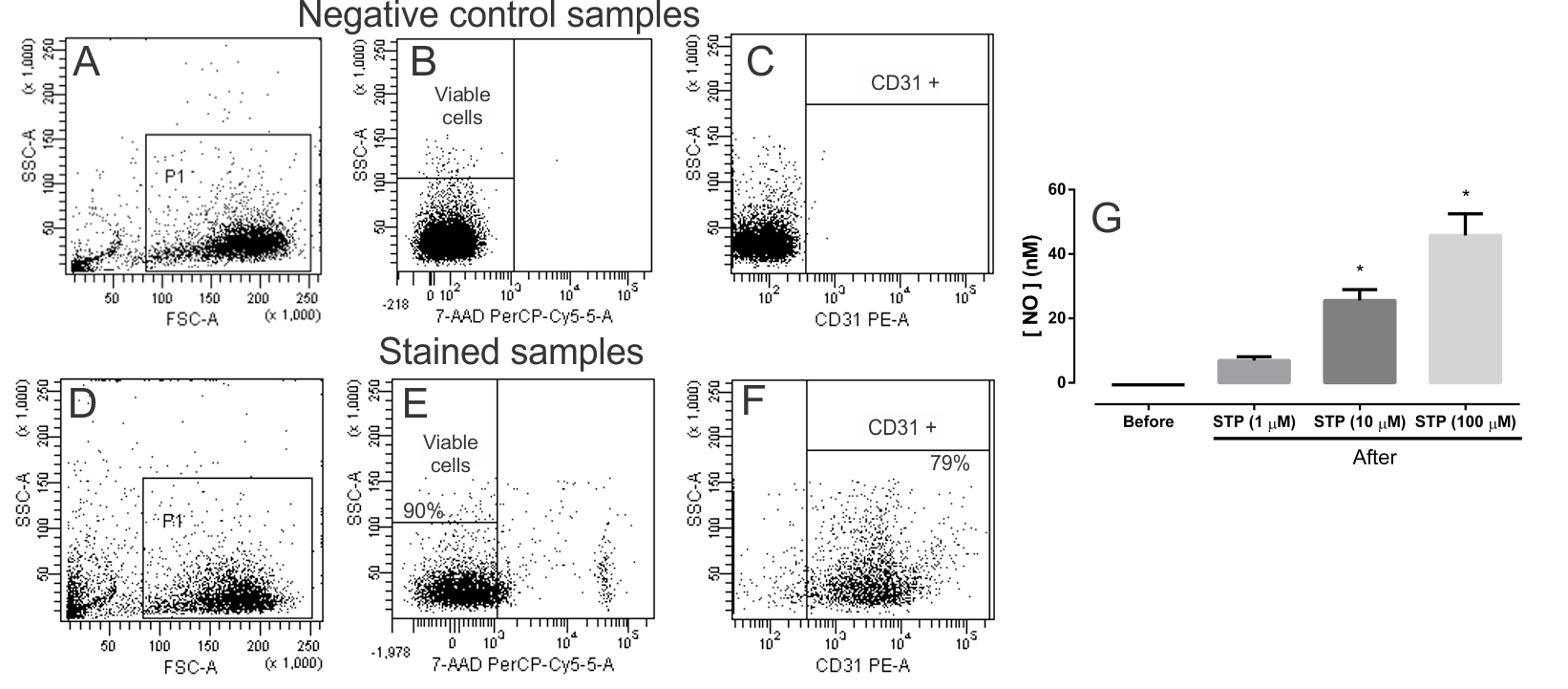

Supplement: Supplementary file 1 [file molecules-23-00253-s001.tif]
